# Sleep disturbance and its association with quality of life among psychiatric nurses in China

**DOI:** 10.7717/peerj.10659

**Published:** 2021-02-17

**Authors:** Li Lu, Ka-In Lok, Qinge Zhang, Ling Zhang, Yifan Xiang, Gabor S. Ungvari, Brian J. Hall, Feng-Rong An, Yu-Tao Xiang

**Affiliations:** 1 Center for Cognition and Brain Sciences, University of Macau, Macau SAR, China; 2 Institute of Advanced Studies in Humanities and Social Sciences, University of Macau, Macao SAR, China; 3 Team IETO, Bordeaux Population Health Research Center, UMR U1219, INSERM, Université de Bordeaux, Bordeaux, France; 4 Kiang Wu Nursing College of Macau, Macau SAR, China; 5 The National Clinical Research Center for Mental Disorders & Beijing Key Laboratory of Mental Disorders, Beijing Anding Hospital & the Advanced Innovation Center for Human Brain Protection, Capital Medical University, Beijing, China; 6 Pui Ching Middle School Macau, Macao SAR, China; 7 Division of Psychiatry, School of Medicine, University of Western Australia, Perth, WA, Australia; 8 University of Notre Dame Australia, Fremantle, WA, Australia; 9 Global and Community Mental Health Research Group, New York University (Shanghai), Shanghai, China

**Keywords:** Sleep disturbance, Nurse, Psychiatry, Quality of life, China

## Abstract

**Background:**

Sleep disturbance is associated with a number of negative adverse outcomes. This study examined the prevalence of sleep disturbance and its association with demographic and clinical characteristics and quality of life (QOL) in psychiatric nurses in China.

**Methods:**

This is a multi-center, cross-sectional study involving 11 psychiatric hospitals in China. Three types of sleep disturbance (difficulty initiating sleep (DIS), difficulty maintaining sleep (DMS) and early-morning awakening (EMA)) and QOL were measured by standardized questions or instruments.

**Results:**

A total of 1,847 psychiatric nurses participated. The overall prevalence of at least one type of sleep disturbance was 71.5% (95% CI [69.3–73.5]); the prevalence of DIS, DMS and EMA was 58.5% (95% CI [56.2–60.8]), 53.7% (95% CI [51.4–56.0]) and 54.6% (95% CI [52.3–56.9]), respectively. Nurses with sleep disturbance had significantly lower QOL in physical (*F*_(1, 1,846)_ = 219.12, *P* < 0.001), psychological (*F*_(1, 1,846)_ = 72.18, *P* < 0.001), social (*F*_(1, 1,846)_ = 37.57, *P* < 0.001) and environmental domains (*F*_(1, 1,846)_ = 95.45, *P* < 0.001). Multivariable logistic regression analyses revealed that shift work (DIS, OR = 1.6, 95% CI [1.28–1.98]; DMS, OR = 1.2, 95% CI [1.001–1.54]; EMA, OR = 1.3, 95% CI [1.02–1.58]) and alcohol use (DIS, OR = 1.8, 95% CI [1.46–2.32]; DMS, OR = 1.8, 95% CI [1.43–2.23]; EMA, OR = 1.7, 95% CI [1.33–2.07]) were positively associated with sleep disturbance, while higher monthly income (DIS, OR = 0.5, 95% CI [0.38–0.75]; DMS, OR = 0.7, 95% CI [0.51–0.98]) was negatively associated with sleep disturbance.

**Conclusion:**

Sleep disturbance was common among nurses in psychiatric hospitals in China, particularly those on shifts and having alcohol use. Health authorities should develop effective measures to reduce risk of sleep disturbance in this population.

## Introduction

Sleep disturbance could lead to a number of adverse outcomes, such as fatigue (Lim et al., 2019), depression (Kalmbach et al., 2017) and suicidal behaviors (Ivbijaro et al., 2019; Porras-Segovia et al., 2019). Epidemiological studies found sleep disturbance was common in healthcare professionals, particularly in nurses (Dong et al., 2017; Giorgi et al., 2018; Weaver et al., 2018). The prevalence of sleep disturbance in nurses varied greatly between different countries and between different specialties. For instance, the prevalence of insomnia was 36% in Brazilian nurses (Silva-Costa, Griep & Rotenberg, 2015), 40.5% in Indian nurses (Khade, Behera & KorradI, 2018), 53.9% in Norwegian nurses (Oyane et al., 2013) and 69.7% in Chinese nurses (An et al., 2016). Nurses working in intensive units (Nazatul et al., 2008) and emergency departments (Dong et al., 2017) were more likely to have sleep disturbance compared to those in other specialties. Sleep disturbance among nurses was associated with higher risk of depression, poor personal relationships with others (Xu et al., 2019; Zhang, Duffy & De Castillero, 2017), burnout (Giorgi et al., 2018) and even medical errors (Park, Lee & Park, 2018). The epidemiology of sleep problems including sleep disturbance is greatly determined by sociocultural and economic factors, therefore the prevalence of sleep disturbance in nurses working in countries with different sociocultural contexts should be examined separately (An et al., 2016).

Quality of life (QOL) is a multidimensional concept and has been used as a comprehensive outcome measure in clinical studies (Felce & Perry, 1995; Hofer et al., 2017). The inverse association between QOL and sleep problems in general population (Lee et al., 2009; Strine & Chapman, 2005) and nurses (Zamanian, Nikeghbal & Khajehnasiri, 2016) have been examined. However, little is known about the relationship between sleep disturbance and QOL among psychiatric nurses in China. To date, only one study involving two psychiatric hospitals examined the prevalence of sleep disturbance (difficulty initiating sleep (DIS): 57.1%; difficulty maintaining sleep (DMS): 56.3% and early-morning wakening (EMA): 57.1%) in nurses in China (An et al., 2016). The lack of QOL measurement and only two study sites limit clinical implications and generalizability of the findings. In 2018, there were 4,098,630 registered nurses in China; of whom, 80,828 (1.97%) worked in psychiatric hospitals (National Health Commission of the PRC, 2019). In order to reduce the negative impact of sleep disturbance on health and work functioning, it is important to examine its epidemiology and relationship with QOL in psychiatric nurses in China.

This study examined the prevalence of sleep disturbance (including DIS, DMS and EMA) among Chinese psychiatric nurses and explored the independent associations of sleep disturbance with demographic characteristics and QOL.

## Materials and Methods

### Settings, subjects and data collection

This is a multi-center and cross-sectional study conducted between October and December 2017. A total of 11 psychiatric hospitals located in 11 provinces were included. The 11 psychiatric hospitals are distributed in the north, east, south, west and central parts of China, which allows us to obtain the perspectives of nurses working in a large geographical area and enhance the representativeness of the survey. All nurses in the participating hospitals were screened and those who were officially employed by hospitals and had worked for at least 1 year were invited to participate in this study. Those who were on leave or were trained elsewhere during the study period were excluded. Questionnaires were distributed to nursing staff personally by trained researchers and were collected at the same day after completion. The assessment was completed on an anonymous and voluntary basis. All nurses involved in this survey provided the informed consent. The study protocol was approved by the Human Research and Ethics Committee of University of Macau and the respective hospitals (BSERE18-APP022-FHS). This study was conducted following the Strengthening the Reporting of Observational Studies in Epidemiology (STROBE) guidelines (Von Elm et al., 2007).

### Assessment tools

Basic demographic characteristics were collected, including: age, gender, working and education years, marital status, monthly income, working in department of psychiatry (yes/no), having children (yes/no), job rank, shift work (yes/no). Following other studies (Xiang et al., 2009), alcohol users were defined as those who answered “yes” to the question: “did you drink alcoholic beverage at least once per month during the past year?”

Sleep disturbance in the past 3 months was assessed by the following three standardized “yes/no” questions (Nogueira et al., 2018): (1) DIS: “nearly every night, it took you two hours or longer to fall asleep”. (2) DMS: “you woke up nearly every night and need an hour or more to get back to sleep”; (3) EMA with inability to return to sleep: “you wake up nearly every morning at least 2 hours earlier than you expected.” The presence of at least one type of sleep disturbance was considered as “having sleep disturbance”.

QOL was assessed by the validated Chinese version of the WHO QOL brief version (WHOQOL-BREF; Cronbach’s alpha: 0.89), which comprised 26 items covering four domains: physical, psychological, social and environmental domains, with a higher score indicating higher QOL (Fang & Hao, 1999; WHO, 1998; Xia et al., 2012).

### Statistical analysis

The normal distributions of continuous variables were measured with the Shapiro–Wilk test. Independent *t*-test, Mann–Whitney *U* test and Chi-square test were conducted to compare socio-demographic and clinical characteristics between nurses with sleep disturbance and those without. Analysis of covariance was performed to compare QOL between the two groups after controlling for socio-demographic variables with group differences in univariate analyses. Multivariable logistic regression analyses were conducted to explore the demographic and clinical correlates of sleep disturbance. Each type of sleep disturbance was entered as dependent variable, while demographic and clinical variables that significantly differed in univariate analyses were entered as independent variables. STATA version 12.0 (Stata Corporation, College Station, TX, USA) were used to perform data analyses with the significance level of 0.05 (two-tailed).

## Results

In total, 2,124 nurses were invited and 1,847 agreed to participate in this study and completed the assessment, giving a response rate of 87.0%. In the entire sample, 71.5% (95% CI [69.3–73.5]; *n* = 1,320) suffered from at least one type of sleep disturbance. Specifically, the prevalence of DIS, DMS and EMA were 58.5% (95% CI [56.2–60.8]; *n* = 1,081), 53.7% (95% CI [51.4–56.0]; *n* = 992) and 54.6% (95% CI [52.3–56.9]; *n* = 1,009), respectively. Of the nurses with sleep disturbances, only 11.5% (152/1,320) ever sought help from doctors.

The comparisons between nurses with sleep disturbances and those without in demographic and clinical characteristics are shown in Table 1. Age, working years, education years and QOL in physical, psychological, social and environmental domains were not normally distributed. Significant differences were found in monthly income, job rank, shift work, and alcohol use. The QOL among Chinese psychiatric nurses were 13.5 (SD = 2.3), 13.0 (SD = 2.6), 13.1 (SD = 3.1) and 11.6 (SD = 2.6) in the physical, psychological, social and environmental domain, respectively. After controlling for the monthly income, job title, shift work and alcohol use, nurses with sleep disturbance had significantly lower QOL in physical health (*F*_(1, 1,846)_ = 219.12, *P* < 0.001), psychological health (*F*_(1, 1,846)_ = 72.18, *P* < 0.001), social domains (*F*_(1, 1,846)_ = 37.57, *P* < 0.001) and in environment domains (*F*_(1, 1,846)_ = 95.45, *P* < 0.001).

**Table 1:** Socio-demographic characteristics of the participants’ and sleep disturbances.

Variables	Total (*n* = 1,847)		No-sleep disturbance (*n* = 527)		Sleep disturbance (*n* = 1,320)		Statistics		
	Mean	SD	Mean	SD	Mean	SD	*Z*	df	*P*
Age (year)	32.4	8.6	32.7	8.5	32.2	8.7	1.49	—^a^	0.136
Working years	11.1	9.4	11.6	9.7	10.9	9.2	1.61	—^a^	0.107
Education years	14.2	2.4	14.3	2.3	14.2	2.5	0.29	—^a^	0.774
Physical QoL	13.5	2.3	14.7	2.1	13.0	2.2	14.66	—^a^	**<0.001**
Psychological QoL	13.0	2.6	13.8	2.5	12.7	2.5	8.59	—^a^	**<0.001**
Social QoL	13.1	3.1	13.8	3.1	12.8	3.1	6.75	—^a^	**<0.001**
Environmental QoL	11.6	2.6	12.6	2.6	11.2	2.5	9.96	—^a^	**<0.001**
	***N***	**%**	***N***	**%**	***N***	**%**	***X***^**2**^	**df**	***P***
Female	1,545	83.7	453	86.0	1,092	82.7	6.73	1^b^	0.086
Married	1,148	62.2	341	64.7	807	61.1	2.04	1	0.153
Monthly income (CNY)							10.49	3	**0.015**
<3,000	301	16.3	76	14.4	225	17.0			
3,000–4,999	554	30.0	145	27.5	409	31.0			
5,000–5,999	632	34.2	180	34.2	452	34.2			
>/=6,000	360	19.5	126	23.9	234	17.7			
Having Children	1,480	80.1	426	80.8	1,054	79.8	0.23	1	0.631
Job rank							5.37	1	**0.02**
Junior nurse	632	34.2	159	30.2	473	35.8			
Senior nurse	1,215	65.8	368	69.8	847	64.2			
Shift work	1,380	74.7	367	69.6	1,013	76.7	10.06	1	**0.002**
Alcohol use	440	23.8	85	16.1	355	26.9	24.05	1^b^	**<0.001**

**Notes:**

a Mann–Whitney test; Bold values: *p* < 0.05.

b Likelihood-ratio chi^2^ test.

Bold values: *p* < 0.05.

CNY, Chinese Yuan; QoL, quality of life.

Multiple logistic regression analyses (Table 2) revealed that nurses on shift work (DIS, OR = 1.6, 95% CI [1.28–1.98]; DMS, OR = 1.2, 95% CI [1.001–1.54]; EMA, OR = 1.3, 95% CI [1.02–1.58]) and those with alcohol use (DIS, OR = 1.8, 95% CI [1.46–2.32]; DMS, OR = 1.8, 95% CI [1.43–2.23]; EMA, OR = 1.7, 95% CI [1.33–2.07]) were more likely to have DIS, DMS and EMA. While those having higher income were less likely to have DIS (OR = 0.5, 95% CI [0.38–0.75]) and DMS (OR = 0.7, 95% CI [0.51–0.98]).

**Table 2:** Logistic regression analyses of different sleep-related disturbances.

Variables	DIS			DMS			EMA		
	OR	*P*	95% CI	OR	*P*	95% CI	OR	P	95% CI
Monthly income (CNY)									
<3,000	1.0	–	–	1.0	–	–	1.0	–	–
3,000–4,999	0.8	0.08	[0.57–1.03]	0.9	0.62	[0.69–1.24]	0.98	0.90	[0.74–1.31]
5,000–5,999	0.7	0.051	[0.55–1.001]	0.8	0.18	[0.61–1.10]	1.01	0.97	[0.75–1.34]
>/=6,000	0.5	**0.001**	[0.38–0.75]	0.7	**0.04**	[0.51–0.98]	0.9	0.38	[0.62–1.20]
Job title									
Junior nurse	1.0	–	–	1.0	–	–	1.0	–	–
Senior nurse	0.9	0.42	[0.74–1.13]	1.03	0.77	[0.84–1.27]	1.02	0.90	[0.82–1.26]
Shift work	1.6	**<0.001**	[1.28–1.98]	1.2	0.050	**[1.001–1.54]**	1.3	**0.03**	[1.02–1.58]
Alcohol use	1.8	**<0.001**	[1.46–2.32]	1.8	**<0.001**	[1.43–2.23]	1.7	**<0.001**	[1.33–2.07]

**Notes:**

Bold values: *p* < 0.05.

CNY, Chinese Yuan; DIS, difficulty initiating sleep; DMS, difficulty maintaining sleep; EMA, early-morning awakening with inability to return to sleep; OR, odds ratio

## Discussion

This was the first multi-center study to examine sleep disturbance and its association with QOL in psychiatric nurses in China. The prevalence of DIS, DMS and EMA were 58.5%, 53.7% and 54.6%, respectively, all of which were negatively associated with QOL. In China, there were 80,828 nurses in psychiatric hospitals in 2018 (National Health Commission of the PRC, 2019). According to the current findings, this translates to approximately 47,284 psychiatric nurses suffering from DIS, 43,405 psychiatric nurses suffering from DMS, and 44,132 psychiatric nurses suffering from EMA.

The prevalence of sleep disturbance (71.5%) in our sample was higher than the corresponding figures of Chinese (up to 63.9%), Brazilian (36%) (Silva-Costa, Griep & Rotenberg, 2015), Indian (40.5%) (Khade, Behera & KorradI, 2018) and Norwegian nurses (53.9%) (Oyane et al., 2013). Our finding was also higher than the corresponding figure in nurses working in internal medicine (62.8%), surgical (62.1%), paediatrics (64.5%) and gynecology and obstetrics departments (63.5%), but similar to the figure in ICUs (71.6%) in China (Dong et al., 2017). In a previous study in China, the prevalence of DIS, DMS and EMA among nurses working in two psychiatric hospitals were 57.1%, 56.3% and 57.1%, respectively (An et al., 2016), which is basically consistent with our results. However, our figure was higher than that among the general population, such as 15.0% in China (Cao et al., 2017), 21.4% in Japan (Kim et al., 2000), 27.6% in Italy (Ohayon & Smirne, 2002). Different cultural, socio-demographic and methodological factors could possibly lead to these prevalence differences. For example, one meta-analysis revealed that the prevalence of sleep disturbances in Chinese healthcare professionals was significantly associated with study year and assessment instruments (Qiu et al., 2020).

Nurses having a higher monthly income were less likely to suffer from sleep disturbance, which confirms previous findings (An et al., 2016). Higher income is usually associated with better general health status and social support (Marmot, 2002) and less living stress (Belle, 1990), which could in turn reduce the risk for sleep disturbance (Åkerstedt et al., 2002; Da Rocha & De Martino, 2010). Alcohol use is associated with increased risk of sleep disturbance in this study, which is consistent with earlier findings (Jefferson et al., 2005; Lydon et al., 2016). The relationships between sleep disturbance and alcohol consumption are bidirectional (Chakravorty, Chaudhary & Brower, 2016; Roehrs & Roth, 2001a). On one hand, alcohol use could affect sleep through its effect on central nervous system (Chakravorty, Chaudhary & Brower, 2016). On the other hand, many persons suffering from insomnia often used alcohol to help them sleep (Ancoli-Israel & Roth, 1999). Previous studies also found a dose relationship between alcohol use and sleep disturbance (Roehrs & Roth, 2001a; Roehrs & Roth, 2001b), but this cannot be examined since the dosage of alcohol was not recorded in this study.

Similar to previous studies (An et al., 2016; Dong et al., 2017; Lee et al., 2007; Shao et al., 2010; Shiffer et al., 2018), there was significant association between sleep disturbance and shift work in this study. Compared to their counterparts on regular work, shift nurses usually slept shorter after night work (Åkerstedt et al., 2002; Tilley et al., 1982). In addition, circadian rhythms could be altered by shift work (Boivin & Boudreau, 2014). Furthermore, work-related stress and pressure are also common in shift working nurses (Wu et al., 2010). All these factors could increase the risk of sleep disturbance. Heavy workload, burnout and workplace violence is common in psychiatric nurses, and these factors are usually associated with lowered QOL (Ibrahim et al., 2016; Kwak et al., 2020). Sleep disturbance may lead to a number of negative outcomes, such as lower job satisfaction (Karagozoglu & Bingol, 2008), emotional disturbance (Lee et al., 2015) and medical errors (Johnson et al., 2014), all of which could further lower QOL (Cimete, Gencalp & Keskin, 2003; Ibrahim et al., 2016). As expected, psychiatric nurses with sleep disturbance reported lower QOL in all domains.

The strengths of this study included multicenter and large sample size. However, several methodological limitations should be noted. First, this is a cross-sectional study, therefore the causality between sleep disturbance and demographic and clinical variables could not be established. Second, some factors possibly associated with sleep disturbance, such as job burnout and turnover (Giorgi et al., 2018), were not analyzed. Finally, only tertiary psychiatric hospitals were involved, thus the findings cannot be generalized to other types of healthcare institutions, such as primary mental health services.

## Conclusions

In conclusion, sleep disturbance is common among psychiatric nurses in China, particularly those on shift work and who use alcohol. Due to the negative impacts on QOL, regular screening and effective treatments should be developed to improve psychiatric nurses’ sleep in China. Previous studies have found that non-pharmacological treatments, such as cognitive behavioural therapy (Cheng & Dizon, 2012) and exercise training (Yang et al., 2012), may effectively improve sleep quality, which could be adopted for those in need.

## Supplemental Information

10.7717/peerj.10659/supp-1 Supplemental Information 1 Raw data.

## Additional Information and Declarations

### Data Availability

The following information was supplied regarding data availability: Raw data are available as a Supplemental File.
